# The Consumer Motivation Scale: A detailed review of item generation, exploration, confirmation, and validation procedures

**DOI:** 10.1016/j.dib.2017.04.054

**Published:** 2017-05-05

**Authors:** I. Barbopoulos, L.-O. Johansson

**Affiliations:** Department of Psychology, University of Gothenburg, Sweden

## Abstract

This data article offers a detailed description of analyses pertaining to the development of the Consumer Motivation Scale (CMS), from item generation and the extraction of factors, to confirmation of the factor structure and validation of the emergent dimensions. The established goal structure – consisting of the sub-goals Value for Money, Quality, Safety, Stimulation, Comfort, Ethics, and Social Acceptance – is shown to be related to a variety of consumption behaviors in different contexts and for different products, and should thereby prove useful in standard marketing research, as well as in the development of tailored marketing strategies, and the segmentation of consumer groups, settings, brands, and products.

**Specifications Table**

**Table:** 

Subject area	*Psychology*
More specific subject area	*Consumer psychology, scale development*
Type of data	*Tables*
How data was acquired	*Survey*
Data format	*Analyzed*
Experimental factors	*A variety of consumer contexts/products, including food, clothes, entertainment, and vacation travel*
Experimental features	*The scale was developed across a variety of contexts and products to ensure a generalizable goal structure*
Data source location	*Gothenburg, Sweden*
Data accessibility	*With the article (+supplementary file for further details)*

**Value of the data**

• Scale development requires a multitude of analyses to be performed, the results of which are often buried in supplementary files, if presented at all. By publishing all the relevant data in the present open access data article, we hope to increase transparency and offer researchers and practitioners alike a detailed overview of the procedures through which the Consumer Motivation Scale (CMS) was developed.
• Researchers and practitioners interested in the CMS, or consumption goals in general, will find a detailed account of the structure and characteristics of influential consumption goals.
• Researchers and students that wish to learn more about scale development may find this article useful as a practical and extensive example of the steps involved in the extraction, confirmation, and validation of psychological factors.

## 1 Data

This data article offers a detailed review of the development of the Consumer Motivation Scale (CMS; [4]). The objective of the research is to establish a structure of sub-goals that form a coherent and practical measurement scale which is:

1. *Integrative* – encompassing not only utilitarian, but also hedonic and normative goals;
2. *Multi-dimensional* – taking potential sub-goals of the three master goals into account;
3. *Context-sensitive* – measuring not only individual, but also situational variance;
4. *General* – applicable to a wide variety of consumption settings and products.

The scale development procedure follows Churchill׳s [12] paradigm for developing marketing constructs. The procedure consists of the following five steps:

1. *Specifying the domain of the construct:* The three master goals and their potential sub-goals are specified and described.
2. *Item generation*: Items were generated based on theories and scales related to the identified sub-goals.
3. *Establishing a factor structure*: The goal structure was explored and purified on Sample 1 A, and confirmed on Sample 1B.
4. *Convergent, discriminant, and construct validity*: Additional data (Sample 2) was collected, thoroughly testing the convergent, discriminant, as well as construct validity of the emergent dimensions.
5. *Criterion-related validity*: Finally, more data (Sample 3) was collected to test criterion-related validity, showing that the dimensions explain choice between alternative products.

The presented data is organized accordingly:

1. *The domain of the construct*
  Table 1. The nine preliminary sub-goals of the gain, hedonic, and normative master goals

**Table 1:** The nine preliminary sub-goals of the gain, hedonic, and normative master goals.

**Goal**	**Sub-goal**	**Underlying motive**
Gain	Value for Money	To get value for money, pay a reasonable price, avoid wasting money^a^
	Quality	To get something of high quality and reliability, that meets one׳s highest expectations^b^
	Function	To get something useful and practical, that serves many purposes^c^
	Safety	To feel safe, calm and prepared for the unforeseen^d^
Hedonic	Pleasure	To get something that satisfies immediate needs, that makes one feel good and happy^e^
	Stimulation	To get something exciting, stimulating or unique, avoid dullness^f^
	Comfort	To get something pleasant and comfortable, avoid hassle and discomfort^g^
Normative	Ethics	To act in accordance with one׳s moral principles and obligations, avoid guilt^h^
	Social Acceptance	To make a good impression, identify with peers, live up to expectations^i^

a Zeithaml [34], Sheth et al. [26], Sweeney and Soutar [29], Lindenberg and Steg [19], Barbopoulos and Johansson [3]

b Zeithaml [34], Dodds et al. [16], Sheth et al. [26], Sweeney and Soutar [29], Lindenberg and Steg [19], Barbopoulos and Johansson [3]

c Sheth, et al. [26]

d Becker [7], Schwartz [25], Rindfleisch and Burroughs [22], Lindenberg and Steg [19], Barbopoulos and Johansson [3]

e Batra and Ahtola [5], Childers et al. [11], Lindenberg and Steg [19]

f Russel [23], Bello and Etzel [8], Watson and Tellegen [33], Ormel et al. [21], Childers et al. [11], Lindenberg and Steg [19], Helm and Landschulze [17], Aluja et al. [1]

g Bello and Etzel [8], Ormel et al. [21], Childers et al. [11], Lindenberg and Steg [19]

h Schwartz [24], Schwartz [25], Dawes and Messick [15], Stern [28], Bamberg and Schmidt [2], Thøgersen [31], Steg et al. [27], Lindenberg and Steg [19], Barbopoulos and Johansson [3]

i McGuire [20], Burnkrant and Cousineau [9], Bearden et al. [6], Cialdini et al. [13], Barbopoulos and Johansson [3]
2. *Item generation*
  Table 2. A list of the adapted and generated items, with items wordings and origin

**Table 2:** A list of the adapted and generated items, with items wordings and origin.

**Item#**	**When I _, it is important that what I choose…**	**Item adapted from:**
VfM1	Is reasonably priced	Sweeney and Soutar [29]
VfM2	Offers value for the money	Sweeney and Soutar [29]
VfM3	Is a good choice considering the price	Sweeney and Soutar [29]
VfM4	Is economical	Sweeney and Soutar [29]
VfM5	Provides good return for the money	Sweeney and Soutar [29]
VfM6	Is not too expensive	–
VfM7	Is not a waste of money	–
Quality1	Is of consistent and high quality	Sweeney and Soutar [29]
Quality2	Is well made (or well performed)	Sweeney and Soutar [29]
Quality3	Has an acceptable standard of quality	Sweeney and Soutar [29]
Quality4	Is first class	–
Quality5	Meets even the highest requirements and expectations	–
Quality6	Has a lasting value	–
Function1	Does not have too limited or short use	Sweeney and Soutar [29]
Function2	Is reliable	Sweeney and Soutar [29]
Function3	Is practical	–
Function4	Is useful	–
Function5	Is functional and fit for purpose	–
Function6	Serves many purposes	–
Safety1	Makes me feel calm and safe	–
Safety2	Makes me feel safe for the future	–
Safety3	Takes consideration of needs that may arise in the future	–
Safety4	Is a good choice in the long-term	–
Safety5	Makes me prepared in case something unforeseen would happen	–
Safety6	Improves my safety or security	–
Pleasure1	Makes me feel good	Childers et al. [11]
Pleasure2	Is enjoyable	Childers et al. [11]
Pleasure3	Is pleasant or enjoyable	Batra and Ahtola [5]
Pleasure4	Makes me want to use it	Sweeney and Soutar [29]
Pleasure5	Appeals to me	–
Pleasure6	Makes me happy and satisfied	–
Pleasure7	Satisfies immediate needs	–
Stimulation1	Is exciting	Childers et al. [11]
Stimulation2	Is interesting	Childers et al. [11]
Stimulation3	Offers diversity	Aluja et al. [1]
Stimulation4	Is not too dull or routine	Aluja et al. [1]
Stimulation5	Gives a unique experience	Bello and Etzel [8]
Stimulation6	Is new or exotic	Bello and Etzel [8]
Stimulation7	Is stimulating	–
Comfort1	Is not too uncomfortable	Childers et al. [11]
Comfort2	Gives relaxation	Bello and Etzel [8]
Comfort3	Gives me rest and recovery	Bello and Etzel [8]
Comfort4	Is not too much bustle or stress	Bello and Etzel [8]
Comfort5	Is not too complicated or strenuous	–
Comfort6	Is smooth and comfortable	–
Comfort7	Makes me feel less stressed out	–
Ethics1	Is consistent with my personal and moral obligations	Steg et al. [27]
Ethics2	Does not make me feel guilt	Steg et al. [27]
Ethics3	Makes me feel like a good person in my own eyes	Steg et al. [27]
Ethics4	Does not violate my principles	Bamberg and Schmidt [2]
Ethics5	Gives me a good conscience	Thøgersen [31]
Ethics6	Is not morally wrong	Thøgersen [31]
Ethics7	Is consistent with my ideals and opinions	–
Social1	Is approved by my friends	Bearden, Netemeyer and Teel [6]
Social2	Is liked by people who are important to me	Bearden et al. [6]
Social3	Is what my friends would expect me to choose	Bearden et al. [6]
Social4	Makes a good impression on people who are important to me	Bearden et al. [6]
Social5	Gives me a sense of belonging with people who are like me	Bearden et al. [6]
Social6	Makes me more alike my role models	Bearden et al. [6]
Social7	Is similar to what people who I identify with choose	Bearden et al. [6]
Social8	Makes me feel accepted	Sweeney and Soutar [29]
Social9	Improves the way I am perceived by people who are Important to me	Sweeney and Soutar [29]
Social10	Is popular among my friends	–

*Note:* Items without references were formulated based on our understanding of the theories related to the given goal. For references to these theories, please see the footnotes to Table 1.
3. *Establishing a factor structure*
  *3.1. Data collection (Sample 1)*
  *3.2 Factor extraction*
    Table 3.2. Factor extraction – The seven-factor structure

**Table 3.2:** Factor extraction – The seven-factor structure.




*Note*: For increased readability, loadings >.5 are bolded while non-significant cross-loadings are colored gray. Items in italics show items which were removed following scale purification.

VfM=Value for Money; Social Ac.=Social Acceptance
  *3.3 Scale purification*
    Table 3.3.1. Items excluded due to insufficient communality (<.5)

**Table 3.3.1:** Items excluded due to insufficient communalities (<.5).

**Original item #**	**Factor**	**Item wording**	**Communality**
Pleasure4	VII	Makes me want to use it	.30
Pleasure7	VII	Satisfies immediate needs	.35
Comfort4	VII	Is not too much bustle or stress	.37
Stimulation3	III	Offers diversity	.41
Stimulation6	III	Is new or exotic	.40
Function1	IV	Does not have too limited or short use	.46
Function6	I	Serves many purposes	.49
Quality3	IV	Has an acceptable standard of quality	.49
    Table 3.3.2. Items excluded due to cross-loadings (>.32 and 50% of main loading)

**Table 3.3.2:** Items excluded due to cross-loadings (2nd loading>.32 and 50% of main loading).

**Original item #**	**Factor**	**Item wording**	**Cross-loading**
Function3	IV	Is practical	85.5%
Comfort7	I	Makes me feel less stressed out	93.0%
Safety4	IV	Is a good choice in the long-term	92.1%
Pleasure6	VII	Makes me happy and satisfied	86.6%
Comfort3	I	Gives me rest and recovery	83.8%
Pleasure5	VII	Appeals to me	70.5%
Function4	VII	Is useful	98.5%
Quality6	I	Has a lasting value	66.9%
Function5	IV	Is functional and fit for purpose	67.4%
    Table 3.3.3. Cronbach’s alpha

**Table 3.3.3:** Cronbach׳s alpha.

**Factor**	**α**
I	.86
II	.92
III	.89
IV	.82
V	.88
VI	.89
VII	.81
  *3.4 Dimension labels*
    Table 3.4. Themes and labels of the seven emergent dimensions

**Table 3.4:** Themes and labels of the seven emergent dimensions.

**Factor**	**Theme**	**Dimension label**
I	Safety, being prepared, future needs	Safety
II	Appearance, gaining approval, fitting in	Social Acceptance
III	Excitement, stimulation, uniqueness	Stimulation
IV	Quality, first class, acceptable standards	Quality
V	Morally correct; per one׳s ideals, principles, conscience	Ethics
VI	Reasonable price, economical, value for money	Value for Money
VII	Comfort, avoid complications and effort	Comfort
  *3.5 Factor confirmation*
    Table 3.5. Confirmatory factor analysis

**Table 3.5:** Confirmatory factor analysis.

	**k**	**χ**^**2**^	**DF**	**χ**^**2**^**/DF**	**Δχ**^**2**^	**ΔDF**	***p***	**RMSEA**	**CFI**
Model 1	1	7879.98	950	8.30	–	–	–	.12	.29
Model 2	3	5354.03	942	5.68	−2525.95	−8	.000	.10	.55
Model 3A	5	4202.28	935	4.49	−1151.75	−7	.000	.08	.67
Model 3B	4	4727.81	939	5.04	−626.22	−3	.000	.09	.61
Model 3C	4	4191.43	939	4.46	−1162.60	−3	.000	.08	.67
Model 4A	7	2398.28	924	2.60	−1793.16	−15	.000	.06	.85
Model 4B	7	1089.49	506	2.15	−1308.79	−418	.000	.06	.89

*Note*: In model 1, all items load on a single general factor. In model 2, items load on their respective master goal (gain, hedonic, or normative), while in models 3A, 3B, 3C, the master goals were split into sub-goals one at a time: in 3A gain was split into the three sub-goals Value for Money, Quality, and Safety; in 3B hedonic was split into Stimulation and Comfort; and in 3C normative was split into Ethics and Social Acceptance. Δ for model 3A, 3B and 3C were calculated in comparison to model 2. Finally, in model 4, all items load according to the PCA. A shorter version (model 4B) was then tested, with the purpose of achieving higher model fit and parsimony. Δ for model 4A was calculated in comparison to the best of model 3A, 3B and 3C (i.e 3C)

k=number of factors
  *3.6 Invariance testing*
    Table 3.6.1. Metric and scalar invariance

**Table 3.6.1:** Metric and scalar invariance.

	**χ**^**2**^	**DF**	**χ**^**2**^**/DF**	**RMSEA**	**CFI**	**ΔCFI**
Configural	4131.53	2530	1.63	.04	.78	
Metric	4293.06	2638	1.63	.04	.77	−.008
**Scalar**	**5069.14**	**2774**	**1.83**	**.04**	**.68**	−**.089**

*Note*: Non-equal models are bolded. Δ was calculated in comparison to the previous model.
    Table 3.6.2. Scalar invariance for each dimension

**Table 3.6.2:** Scalar invariance for each dimension.

	**χ**^**2**^	**DF**	**χ**^**2**^**/DF**	**RMSEA**	**CFI**	**ΔCFI**
Metric	4293.06	2638	1.63	.04	.77	
VfM	4375.34	2658	1.65	.04	.76	−.008
**Quality**	**4411.63**	**2658**	**1.66**	**.04**	**.76**	**−.013**
**Safety**	**4477.24**	**2658**	**1.68**	**.04**	**.75**	**−.022**
**Stimulation**	**4503.30**	**2658**	**1.69**	**.04**	**.74**	**−.026**
**Comfort**	**4423.08**	**2654**	**1.67**	**.04**	**.75**	**−.016**
Ethics	4327.59	2658	1.63	.04	.77	−.002
Social Ac.	4365.66	2658	1.64	.04	.76	−.007

*Note*: Non-equal dimensions are bolded. Δ was calculated in comparison to the invariant metric model.

VfM=Value for Money; Social Ac.=Social Acceptance.
    Table 3.6.3. Scalar invariance for each item in Quality, Safety, Stimulation, and Comfort

**Table 3.6.3:** Scalar invariance for each item in Quality, Safety, Stimulation, and Comfort.

	**χ**^**2**^	**DF**	**χ**^**2**^**/DF**	**RMSEA**	**CFI**	**ΔCFI**
Metric	4293.06	2638	1.63	.04	.77	
Quality1	4342.16	2642	1.64	.04	.76	−.006
Quality2	4325.29	2642	1.64	.04	.77	−.004
Quality3	4308.09	2642	1.63	.04	.77	−.001
Quality4	4301.89	2642	1.63	.04	.77	.000
Quality5	4314.14	2642	1.63	.04	.77	−.002
**Safety1**	**4398.45**	**2642**	**1.67**	**.04**	**.76**	**−.014**
**Safety2**	**4392.01**	**2642**	**1.66**	**.04**	**.76**	**−.013**
**Safety3**	**4375.08**	**2642**	**1.66**	**.04**	**.76**	**−.011**
**Safety4**	**4398.12**	**2642**	**1.67**	**.04**	**.76**	**−.014**
Safety5	4359.77	2642	1.65	.04	.76	−.008
Stimulation1	4361.66	2642	1.65	.04	.76	−.009
**Stimulation2**	**4402.37**	**2642**	**1.67**	**.04**	**.76**	**−.014**
Stimulation3	4327.80	2642	1.64	.04	.77	−.004
**Stimulation4**	**4383.14**	**2642**	**1.66**	**.04**	**.76**	**−.012**
**Stimulation5**	**4421.29**	**2642**	**1.67**	**.04**	**.75**	**−.017**
Comfort1	4374.40	2642	1.66	.04	.76	−.010
Comfort2	4370.11	2642	1.65	.04	.76	−.010
Comfort3	4326.79	2642	1.64	.04	.77	−.004
Comfort4	4330.58	2642	1.64	.04	.77	−.004

*Note*: Δ was calculated in comparison to the invariant metric model. Non-equal items are bolded. The item numbers correspond to the numbers in the final version of the CMS (see Table 3.7)
    Table 3.6.4. Structural and strict invariance

**Table 3.6.4:** Structural and strict invariance.

	**χ**^**2**^	**DF**	**χ**^**2**^**/DF**	**RMSEA**	**CFI**	**ΔCFI**
Partial scalar	4520.09	2718	1.66	.04	.75	
**Structural**	**4831.25**	**2746**	**1.76**	**.04**	**.71**	**−.039**
**Strict**	**5305.49**	**2882**	**1.84**	**.04**	**.66**	**−.047**

*Note*: Δ was calculated in comparison to the previous model. Non-equal models are bolded.
  *3.7 Finalized version of the Consumer Motivation Scale (CMS)*
  Table 3.7. The Consumer Motivation Scale (CMS)

**Table 3.7:** The Consumer Motivation Scale (CMS).

	**Item #**	**What matters the most to you when you _?**
Value for Money	VfM1	**Reasonable price:** the product should be reasonably priced
	VfM2	**Not too expensive:** the product should not be too expensive
	VfM3	**Economy:** the product should be economical
	VfM4	**Value for money:** I should get a lot for the price I pay
	VfM5	**Not wasteful:** the product should not be a waste of money
Quality	Quality1	**Quality:** the product should be of consistent and high quality
	Quality2	**First class:** the product must be of the highest class
	Quality3	**Well made:** the product should be well-made or well-performed
	Quality4	**Fulfills expectations:** the product should fulfill even my highest requirements and expectations
	Quality5	**Reliability:** the product should be reliable (I should know what I get)
Safety	Safety1	**Security:** the product should provide a prolonged and persistent feeling of security
	Safety2	**Safe and secure:** the product should feel safe and secure
	Safety3	**Preparation:** the product should make me well-prepared in case something unforeseen happens
	Safety4	**Calm and safe:** the product should make me feel calm and safe
	Safety5	**Future needs:** Needs that may arise in the future should be taken into consideration
Stimulation	Stimulation1	**Exciting:** the product should be exciting
	Stimulation2	**Stimulating:** the product should be stimulating
	Stimulation3	**Avoid boredom:** It is important that the product is not too boring or routine
	Stimulation4	**Unique:** the product should be unique (or give many unique experiences)
	Stimulation5	**Interesting:** the product should be interesting
Comfort	Comfort1	**Smoothness:** the product should be smooth and comfortable
	Comfort2	**Avoid inconvenience:** the product should not be too inconvenient
	Comfort3	**Avoid hassle:** the product should not be too complicated or strenuous
	Comfort4	**Pleasure:** the product should be pleasant and agreeable
Ethics	Ethics1	**Not morally wrong:** the product should not be morally wrong
	Ethics2	**Principle:** the product should not violate my principles
	Ethics3	**Obligations:** the product should be compatible with my personal and moral obligations
	Ethics4	**Ideals and opinions:** the product should be compatible with my ideals and opinions
	Ethics5	**Good conscience:** the product should give me a good conscience
Social Ac.	Social1	**Friends’ approval:** the product should be approved by my friends
	Social2	**Popularity:** the product should be popular in my circle of friends
	Social3	**Friends’ expectations:** the product should not go against my friends’ expectations of me
	Social4	**Good impression:** the product should make a good impression on people who are important to me
	Social5	**Liked:** the product should be liked by people who are important to me

*Note*: The dimension labels and the item # should not be visible when used in a questionnaire, and the items should be presented in randomized order.

Social Ac.=Social Acceptance
4. *Convergent, discriminant, and construct validity*
  *4.1. Data collection (Sample 2)*
    Table 4.1. The target information types, upgrade packages, and reference scales for the seven dimensions of the CMS

**Table 4.1:** The target information types, upgrade packages, and reference scales for the seven dimensions of the CMS.

	**Information type**	**Upgrade package**	**Reference scale**
VfM	I seek rebate and deals	Get 600 SEK discount per person [approx. €60]	PERVAL-price Sweeney, Soutar [29]
Quality	I seek information about hotel classifications and quality standards	Change to a 4-star hotel with excellent quality and great feeling for details, in everything from interior design to service	PERVAL-quality Sweeney, Soutar [29]
Safety	I seek information regarding insurances, safety, unrest	Get an extra travel insurance without deductible, with compensation for lost or damaged goods, free health care and cancellation protection	Security value Schwartz[25]
Stimulation	I seek information about activities, sights and experiences	Change to an ‘adventure hotel’ where bungee jumping, rafting, kite surfing, mountain climbing, diving and other exciting activities are arranged	Sensation-seeking Aluja et al., [1]
Comfort	I try to find out what׳s available at the hotel or in close vicinity to the hotel	Upgrade flight seat to 1st class and change to an extra comfortable room. You will also get access to the hotel spa and relax facilities with Jacuzzi, sauna and various spa and massage treatments	Restful experience Bello, Etzel [8]
Ethics	I seek information about environmental standards, environmental impacts and carbon emission	Change to an environmentally certified hotel and fly with an airplane which emits about 30% less carbon dioxide per passenger	Universalism value Schwartz [25]
Social Ac.	I ask my friends and acquaintances about their opinions and recommendations	Change to a hotel which has 4.5/5 user rating and lies in an area which recently has been selected "One of the summers׳ trendiest travel destination" by a panel of well-known fashion and travel magazines	CSII Bearden et al., [6]

VfM=Value for Money; Social Ac.=Social Acceptance
  *4.2. Convergent validity*
    Table 4.2.1. Factor loadings for item 1-5 in each dimension, as well as average communalities and Cronbach’s alpha

**Table 4.2.1:** Factor loadings for item 1–5 in each dimension, as well as average communalities and Cronbach׳s alpha.

	**VfM**	**Qua.**	**Saf.**	**Sti.**	**Com.**	**Eth.**	**Soc.**
Item #1	.83	.82	.80	.83	.79	.87	.85
Item #2	.82	.80	.77	.79	.82	.86	.78
Item #3	.78	.71	.76	.79	.75	.87	.81
Item #4	.73	.65	.75	.77	.65	.78	.80
Item #5	.73	**.44**	.66	.74	–	.66	.75
Average loading	.78	.68	.75	.78	.75	.81	.80
Average communality	.67	.64	.65	.67	.64	.71	.67
Cronbach׳s alpha	.86	.81	.85	.86	.80	.88	.85

*Note*: Values that fail to surpass the thresholds are bolded.

VfM=Value for Money; Qua.=Quality; Saf.=Safety; Com.=Comfort; Eth.=Ethics; Soc.=Social Acceptance
    Table 4.2.2. Component reliability (CR) and average extracted variance (AVE)

**Table 4.2.2:** Component reliability (CR) and average extracted variance (AVE).

	**CR**	**AVE**
Value for Money	.86	.55
Quality	.80	**.45**
Safety	.85	.53
Stimulation	.84	.52
Comfort	.79	.50
Ethics	.88	.59
Social Acceptance	.85	.53

*Note*: Values that fail to surpass the thresholds are bolded.
    Table 4.2.3. Bivariate correlation coefficients for each dimension of the CMS and its reference scale

**Table 4.2.3:** Bivariate correlation coefficients for the CMS dimensions and their reference scales.

**CMS dimension**	***r***	***p***	**Reference scale**
Value for Money	.36	.000	PERVAL-price
Quality	.67	.000	PERVAL-quality
Safety	.32	.000	Family security
Stimulation	.46	.000	Sensation-seeking
Comfort	.67	.000	Restful experience
Ethics	.45	.000	Universalism
Social Acceptance	.53	.000	CSII
  *4.3. Discriminant validity*
    Table 4.3.1. Cross-loadings by item number (1-5) and dimension

**Table 4.3.1:** Cross-loadings as % of the main loading, by item number (1–5) and dimension.

	**VfM**	**Qua.**	**Saf.**	**Sti.**	**Com.**	**Eth.**	**Soc.**
Item #1	6.1%	11.5%	14.2%	8.9%	11.6%	15.3%	10.5%
Item #2	22.4%	23.0%	12.2%	13.7%	2.3%	7.8%	15.6%
Item #3	17.5%	11.5%	13.7%	12.6%	11.1%	8.0%	8.4%
Item #4	25.8%	38.2%	12.0%	20.8%	19.2%	18.7%	5.9%
Item #5	12.1%	**56.3%**	21.2%	6.9%	–	26.0%	13.7%

VfM=Value for Money; Qua.=Quality; Saf.=Safety; Com.=Comfort; Eth.=Ethics; Soc.=Social Acceptance
    Table 4.3.2. Component correlations between the dimensions

**Table 4.3.2:** Component correlations between the dimensions.

	**VfM**	**Qua.**	**Saf.**	**Sti.**	**Com.**	**Eth.**	**Soc.**
VfM	–						
Qua	.25	–					
Saf	.41	.35	–				
Sti	.06	.32	.28	–			
Com	.46	.41	.45	.18	–		
Eth	.27	.32	.39	.35	.26	–	
Soc	.13	.19	.33	.17	.20	.18	–

VfM=Value for Money; Qua.=Quality; Saf.=Safety; Com.=Comfort; Eth.=Ethics; Soc.=Social Acceptance
    Table 4.3.3. Average variance extracted (AVE), maximum shared variance (MSV), and average shared squared variance (ASV)

**Table 4.3.3:** Average variance extracted (AVE), maximum shared variance (MSV), and average shared squared variance (ASV).

	**AVE**	**MSV**	**ASV**
VfM	.55	.29	.12
Qua	.45	.20	.06
Saf	.53	.29	.11
Sti	.52	.21	.11
Com	.50	.22	.16
Eth	.59	.20	.07
Soc	.53	.22	.12

VfM=Value for Money; Qua.=Quality; Saf.=Safety; Com.=Comfort; Eth.=Ethics; Soc.=Social Acceptance
    Table 4.3.4. Target correlations vs. unrelated correlations

**Table 4.3.4:** Target correlations vs. unrelated correlations.

	**VfM**	**Qua.**	**Saf.**	**Sti.**	**Com.**	**Eth.**	**Soc.**
Target *r*	.36	.67	.32	.49	.67	.45	.58
Avg. unrelated *r*	.24	.38	.41	.11	.39	.23	.17
Z	1.48	4.61	−1.17	4.78	4.48	2.81	5.51
*p*	.139	.000	.242	.000	.000	.005	.000

VfM=Value for Money; Qua.=Quality; Saf.=Safety; Com.=Comfort; Eth.=Ethics; Soc.=Social Acceptance
  *4.4. Construct validity*
    Table 4.4.1. Standardized regression coefficients for the dimensions in the CMS and the reference scales (Ref.) for information search behaviors (Info.) and upgrade preferences (Pref.). The hypothesized relations are identified in the bolded diagonal (CMS), and in the bolded column (reference scales)

**Table 4.4.1:** Standardized regression coefficients for the dimensions in the CMS and the reference scales (Ref.) for information search behaviors (Info.) and upgrade preferences (Pref.). The hypothesized relations are identified in the bolded diagonal (CMS), and in the bolded column (reference scales).

*Note*: For increased readability, hypothesized relations are in bold, and non-significant relations are colored gray.

VfM=Value for Money; Qua.=Quality; Saf.=Safety; Sti.=Stimulation; Con.=Convenience; Eth.=Ethics; Soc.=Social Acceptance; Envir.=Environmental*p<.05.**p<.01.***p<.001.
    Table 4.4.2. Comparison of target correlations for the CMS versus the reference scales (Ref.)

**Table 4.4.2:** Comparison of target correlations for the CMS versus the reference scales (Ref.).

	**CMS**		**Ref.**	
	***r***		***r***	**Z**
Deals	.36	=	.35	<1
Discount	.17	=	.11	<1
Classification	.59	=	.59	<1
4-star	.48	=	.44	<1
Safety	.42	>	.11	3.79***
Travel insurance	.30	=	.35	>−1
Activities	.34	>	.09	2.96**
Adventure	.31	<	.50	-2.57*
Availability	.47	>	.29	2.37*
Comfort	.35	=	.29	<1
Envir. impact	.49	=	.35	1.92
Envir. certificate	.51	=	.51	<1
Friends	.28	=	.16	1.42
Popular	.31	=	.42	−1.43

Envir = Environmental.

* *p*<.05.

** *p*<.01.

*** *p*<.001.
  *5. Criterion-related validity*
  *5.1. Data collection (Sample 3)*
  *5.2. Criterion-related validity*
    Table 5.2.1. Observed and predicted choices per the binary logistic regression.

**Table 5.2.1:** Observed and predicted choices per the binary logistic regression.

		**Predicted**	
		**Green**	**Regular**
**Observed**	**Green**	92	35
	**Regular**	31	98
			
Correct green	92		
Correct regular	98		
Correct total	190	74.2%	
Incorrect total	66	25.8%
    Table 5.2.2. Logistic regression coefficients and odds ratio for the dimensions of the CMS, as well as pseudo R2 for the whole model.

**Table 5.2.2:** Logistic regression coefficients and odds ratio for the dimensions of the CMS, as well as pseudo R^2^ for the whole model.

	**B**	**Wald**	***p***	**Odds ratio**
Value for Money	−1.16	24.32	.000	−3.19
Quality	−.15	.38	.538	−1.16
Safety	−.34	3.16	.076	−1.40
Stimulation	.61	9.29	.002	1.84
Comfort	−.04	0.03	.856	−1.04
Ethics	1.20	34.99	.000	3.32
Social Acceptance	−.27	3.60	.058	−1.31
Constant	.64	.46	.496	1.90
-2 Log likelihood	264.71			
Cox & Snell R Square	.30			
Nagelkerke R Square	.40

Please see [4] for the theoretical background and discussion related to the conclusions and implications of the data and the CMS, and see the supplementary file for further details pertaining to the data presented in this article.

## 2 Experimental design, materials and methods

2.1. The domain of the construct

The Consumer Motivation Scale (CMS; [4]) builds on the goal-framework developed by Lindenberg and Steg [19], in which three overarching “master” goals are identified and described, namely: the *gain goal* (“to guard or improve one׳s resources”; [19], p. 119), the *hedonic goal* (“to feel better right now”; [19], p. 119) and the *normative goal* (“to act appropriately”; [19], p. 119). The three master goals are assumed to consist of several sub-goals, that in turn are related to means and behaviors.

In recent exploratory research by the authors of the present data article [3], the three higher-order goals were shown to be represented by multiple distinct sub-goals, as the gain goal emerged as two distinct dimensions, one dealing with thrift, and the other with financial security. Likewise, the normative goal emerged as two separate dimensions, one dealing with ideals and moral norms, and the other with social status and fitting in. It was concluded that. It was concluded that the hedonic master goal can likely be represented by multiple sub-goals as well, and that the dimensionality of the master goals should be examined further. To this end, an in-depth literature review was conducted with the purpose of identifying relevant theories and measures related to each of the three master goals. Based on this review, nine preliminary sub-goals were identified and described (Table 1).

2.2. Item generation

Items were adapted or generated, based on a selection of established theories and scales related to the nine preliminary sub-goals. Generated items were formulated based on our understanding of the theories related to the given goal, while adapted items were based on the content of established scales (see Table 2 for references). To get information about the active goals, the scale is introduced with the statement “When I _, it is important that what I choose…” (where the blank is replaced by a suitable reference to the product under study, e.g. “shop for clothes”), followed by the list of items, represented as the continuation of that statement (e.g. “… is not too expensive”).

## 3 Establishing a factor structure

### 3.1 Data collection (Sample 1)

Nine-hundred eighty-seven respondents were recruited from a general population research panel at the University of Gothenburg, Sweden. The CMS was presented with the question “When I _, it is important that what I choose…” (where the blank was replaced by one of the following: “shop for food”, “shop for clothes”, “shop for something that is entertaining or amusing”, “spend money on travel”, or “look for housing”), followed by the list of items, represented as the continuation of that question (e.g. “… is not too expensive”). The participants were asked to rate the importance of the 63 items in their respective context, on a seven-point scale, ranging from 0 (not at all important) to 6 (extremely important). The sample was then randomly split into two halves, with exploratory analyses performed on the former (Sample 1 A), and confirmatory on the latter (Sample 1B).

### 3.2 Factor extraction

Principal component analysis (PCA) was conducted on Sample 1 A (N = 496) to find the structure with the highest explained variance without signs of over-extraction. Over-extraction was defined as a structure with at least one factor made up of fewer than three main loading items [14], while a main-loading item was defined as an item with a factor loading of .5 or greater, and that does not have a cross-loading which amounts to at least .32 and >50% of the main loading [30].

The conditions for PCA were met in that KMO is larger than .6 (.923) and Bartlett׳s *p* is significant (*p*<.001; [18]). An oblique rotation was used since the dimensions are assumed to be naturally correlated.

Of the structures that meet the set criteria, the seven-factor structure is the structure with the highest explained variance (59.9%; Table 3.2.1). In comparison, the eight-factor structure has one factor that is made up of only two items, none of which load greater than .5.

### 3.3 Scale purification

The emergent structure was then purified by removing weak and cross-loaded items one by one, recalculating the communalities and factor loadings after each removal, with the following criteria being used for exclusion:

1. *Insufficient communality (*Table 3.3.1*)*: Communality *<* .5 [14]; or:
2. *Significant cross-loading (*Table 3.3.2*)*: Secondary loading that amounts to at least .32 and 50% of the main loading [15], [31].

One additional item was removed from factor III: Comfort3 “gives relaxation”. The decision was based on content rather than communalities or cross-loadings. The other items in factor III are about stimulation and excitement, and so we opted to exclude this item to keep the factor relatively clean. The resulting 45-item scale consists of seven distinct factors, explaining a total of 65.7% of the variance.

Cronbach׳s alpha was calculated to ensure that the established dimensions are sufficiently reliable (i.e. >.7, or preferably >.8; [18]; Table 3.3.3).

### 3.4 Dimension labels

The seven emergent dimensions correspond to seven of the nine preliminary dimensions, and the labels were therefore applied accordingly. Factor I is entirely made up of items from the preliminary Safety dimension and the label was therefore retained. Likewise, factor II is made up of items from the Social Acceptance dimension, factor V of items from the Ethics dimension, and VI of items from the Value for Money dimension. For the remaining three factors, III, IV, and VII, one of the preliminary dimensions is clearly focal in terms of number of items as well as factor loadings, with a maximum of one or two items from another dimension. The preliminary labels were therefore retained for them as well. Only Function and Pleasure did not emerge as distinct factors.

### 3.5 Factor confirmation

Confirmatory factor analysis was performed on sample 1B (*N*=491; Table 3.5) to confirm the emergent factor structure. A null model, in which all variables were assumed to be uncorrelated, was compared to four specified models, with increasing levels of separation between the dimensions. In model 1, all items load on a general factor, in model 2, all items load on factors representing the master goals: gain (Value for Money, Quality and Safety items), hedonic (Stimulation and Comfort), and normative (Ethics and Social Acceptance), in models 3A, 3B, and 3C, the master goals were split into sub-goals one at a time: in 3A gain was split into the three sub-goals Value for Money, Quality, and Safety; in 3B hedonic was split into Stimulation and Comfort; and in 3C normative was split into Ethics and Social Acceptance. Finally, in model 4, all items load according to the PCA. A shorter version (model 4B) was then tested, with the purpose of achieving higher model fit and parsimony. In this model, items per dimension was reduced to a maximum of five, based on factors loadings as well as content (in general, we wanted strong loadings while maintaining varied content), resulting in a 34-item model.

Model fit improved significantly for each model (*p*<.001), with model 4 A and 4B both reaching satisfactory fit in terms of χ^2^/DF (<3) and RMSEA (<.10), while model 4B approach satisfactory CFI (.89; >.9 is preferable, but these cut-offs are not clear-cut).

### 3.6 Invariance testing

The scale was tested for invariance on Sample 1B, first for factor loadings (metric invariance) and intercepts (scalar invariance), and then for factor means (structural invariance) and residuals (strict invariance). While the model should be invariant on the metric and scalar levels to allow for meaningful comparisons between contexts, it is assumed that the model is *not* invariant on the structural level, as the means are expected to vary from context to context, and we do not require our model to be invariant on the strict level, even though it would be preferable. As the name applies, strict invariance is simply not feasible for most models [32].

First, a baseline configural model was defined, in which the contexts were specified, but all parameters remained unconstrained across the contexts. Each subsequent model then builds on the previous model, but with added constraints for that level of measurement; factor loadings were constrained equal for the metric model, factor loadings and intercepts for the scalar model, factor loadings, intercepts, and means for the structural model, and finally, factor loadings, intercepts, means, and residuals for the strict model.

As a general criterion, a model was considered invariant if ΔCFI is not below −.01 in comparison to the previously accepted model [10]. If invariance does not hold for a model (i.e. ΔCFI is below −.01), then the test is repeated for each dimension, and if invariance does not hold for a dimension, the test is repeated for each item in that dimension, thus identifying the source of the non-equality. For partial invariance to hold, a dimension should have at least two invariant items.

As the scalar model has a ΔCFI below −.01, invariance of intercepts is tested for each dimension on the scalar level of measurement (Table 3.6.2.).

As ΔCFI is below −.01 for Quality, Safety, Stimulation, and Comfort, scalar invariance is tested for each item in these dimensions (Table 3.6.3.).

All items are invariant for the Quality and Comfort dimensions, while Stimulation has at least two invariant items, thus meeting the criterion for partial invariance. Unfortunately, Safety only has one invariant item, and is therefore not sufficiently invariant on the scalar level. Since situational fluctuations in intercepts could influence the mean, situational differences in this dimension should therefore be interpreted carefully. However, two things should be noted: first, ΔCFI is not extreme, ranging from −.011 to −.014, and second, contexts that are very different from each other increase the likelihood that a model will fail to be invariant. The contexts in the present study were purposefully chosen as they represent different areas of consumption, and should therefore be considered a harsh test of invariance. For instance, when the housing context is dropped from the model – leaving food, clothes, entertainment, and travel – all five Safety items achieve satisfactory invariance on the scalar level, with ΔCFI ranging from −.001 to −.005. Thus, situational differences in the Safety dimension should be interpreted carefully across contexts that are very different from each other (e.g. food vs. housing), but it is unlikely that this would be problematic in most scenarios.

For the final test, the partially invariant scalar model, in which the constraints for the non-equal intercepts were relaxed, was compared to the constrained structural (factor means) as well as strict (residuals) models (Table 3.6.4.).

As expected, ΔCFI is below −.01 for the structural model, which suggests that the means of the dimensions do indeed vary significantly across contexts. These results were replicated in a MANOVA: all dimensions vary significantly (F range from 6.3 to 39.64, all significant at *p*<.001, and *η2* range from .05 to .26), except Ethics (F [4, 441] = 1.32, *p*=.261, *η2* = .012). It is not within the scope of the present article to further test variations across contexts, please see [5], for a series of experiments testing the situational activation of the sub-goals across functional, hedonic, and social situations.

Invariance of residuals (strict invariance) is not supported across these contexts.

### 3.7 Finalized version of the Consumer Motivation Scale (CMS)

In the finalized 34-item version of the scale, the items are introduced by the question “What matters the most to you when you _?” (where the blank is replaced by a suitable reference to the product under study, e.g. “shop for clothes”), followed by the list of items, representing statements that answer the question. Note that the item wordings were modified slightly from previous versions, in order to improve clarity and allow it to be better tied to the context at hand. For instance, "is reasonably priced" was changed to "**Reasonable price:** the product should be reasonably priced" (where “the product” can be changed freely to suit the context, although this is optional). The participants then rate to what extent each statement is important to them in the given context, for instance rated on a six-point scale, from 0 (not at all important) to 5 (extremely important).

## 4 Convergent, discriminant, and construct validity

### 4.1 Data collection (Sample 2)

Two-hundred fifty-five respondents were recruited from a pool of voluntary research participants at the University of Gothenburg, Sweden. Participants were asked to what extent they search for different kinds of information before they decide where to go for vacation (rated on a six-point scale ranging between 0 [not at all] to 5 [to a very high degree]), and which of seven hypothetical travel package upgrades they prefer (rated on a five-point scale ranging from 1 [least preferred] to 5 [most preferred]). One information search behavior and one hypothetical travel package was formulated for each dimension of the CMS.

The final version of the CMS was used in this study (see Table 3.7), introduced with the question “What matters the most to you when you choose among vacation trips?” (note that “the product” part of the item wordings in Table 3.7 was changed to “the vacation trip” to suit the context), rated on a six-point scale, from 0 (not at all important) to 5 (extremely important).

The questionnaire also contained a selection of similar scales (from here on referred to as “reference scales”); one reference scale was selected for each dimension. For Value for Money and Quality, items from the PERVAL dimensions price and quality [29] were included and rated on the same scale as the CMS. For Safety, Schwartz׳s [25] security value type was selected, rated on a six-point scale ranging from 0 (not at all important) to 5 (extremely important). The sensation-seeking scale (SSS; [1]) was chosen as reference to Stimulation, rated on a six-point scale ranging from 0 (does not apply at all) to 5 (applies precisely). The restful experience dimension reported by Bello and Etzel [8] was used for Comfort, and was rated on the same scale as the CMS.

The universalism value type [25] was chosen for Ethics, rated on a six-point scale ranging from 0 (not at all important) to 5 (extremely important), and for Social Acceptance, consumer susceptibility to interpersonal influences (CSII; [6]) was chosen, rated on a six-point scale ranging from 0 (does not apply at all) to 5 (applies precisely).

### 4.2 Convergent validity

Convergent validity was tested on Sample 1 A by recalculating the PCA on the confirmed 34-item scale. The factor loadings should be at least > .5 (but preferably > .7; [18]), a criterion met by all items except one. The internal consistency of the dimensions is satisfactory, with Cronbach׳s alpha ranging from .81 to .88 (> .7 is commonly regarded as acceptable).

Several measures were calculated on Sample 1B to test convergent validity (Table 4.2.2). According to Hair et al. [18], convergent validity is supported if average variance extracted (AVE) is greater than .5 and if composite reliability (CR) in turn is greater than AVE. These criteria were met for all dimensions except Quality (AVE=.45). Low AVE may indicate low factor loadings, however, since four of the five factor loadings are significant on Sample 1A as well as 1B, this is not considered problematic for the dimension as a whole.

Bivariate correlations were calculated for each dimension of the CMS and its reference scale on Sample 2 (N = 255). All dimensions correlate positively and significantly with their respective reference scale. Note that a single item from the security value type, namely “Family security”, was chosen as reference scale for Safety due to insufficient Cronbach׳s alpha when the items were combined (.60). Note that as there are a few shared items between the CMS and the reference scales, whenever the CMS dimension and the given reference scale is correlated, the overlapping items were excluded from the CMS to avoid inflated correlations.

### 4.3 Discriminant validity

To test discriminant validity, the factor loadings and component correlations from the recalculated PCA were examined once more, this time to check for remaining cross-loadings and excessive overlap. There is only one remaining cross-loading, and correlations between components are not excessive (<.7).

Several measures were calculated on Sample 1B to test discriminant validity. According to Hair et al. [18], discriminant validity is supported if the average variance extracted (AVE) is greater than the maximum shared squared variance (MSV) as well as the average shared squared variance (ASV), criteria met by all dimensions.

To test discriminant validity on Sample 2, the “target” bivariate correlation coefficients in Table 4.2.3 were compared to the average correlation between a given dimension of the CMS and the six “unrelated” scales (i.e. the reference scales of the other dimensions). Fisher׳s r-to-Z transformation was calculated for significance testing.

The target correlations were significantly stronger than the average unrelated correlations for all dimensions except Value for Money and Safety. For Value for Money, the non-significant Z likely depends on the relatively weak correlation between this dimension and its reference scale (.36). Since Value for Money performs well on the previous tests of convergent as well as discriminant validity, this is not seen as problematic. For Safety, the negative Z value likely depends on the relatively strong correlation with the unrelated scales (.41). Excessive correlations between the dimensions were not observed in previous contexts, and the overlap is therefore concluded to be contextual. A high correlation between, for instance, Safety, Comfort, and Quality, may in fact be expected in a travel context, as few vacation goers would consider a vacation package that is unsafe and uncomfortable to be of high quality.

### 4.4 Construct validity

Construct validity was tested on Sample 2 by performing a series of regression analyses with the dimensions of the CMS as independent variables, and the 14 target constructs as dependent variables. For comparison, a parallel model was defined in which the CMS was replaced by the reference scales. All target regression coefficients for the CMS were statistically significant at *p*<.05. The reference model performed worse in this regard, as five coefficients out of 14 were non-significant.

Also, bivariate correlations were calculated for each dimension of the CMS/reference scale on the one hand, and the seven target constructs on the other. The coefficients of the CMS were then compared to the coefficients of the reference scales, using Fisher׳s r-to-Z transformation. The coefficients of the CMS were significantly stronger in three cases, and weaker in one case.

Note that due to limitations in size and format of the questionnaire, the reference scales could not always be included in full or exactly as intended by their authors (the stimulation-seeking scale, for instance, consists of 40 items alone). The comparison between the CMS and the bundle of reference scales should therefore not be considered a test of the reference scales themselves, but rather as a test of the principle of bundling different scales to represent a multi-dimensional measure. The difficulty in accomplishing this is in itself an advantage of the CMS.

## 5 Criterion-related validity

### 5.1 Data collection (Sample 3)

Two-hundred fifty-six participants were recruited in a class room environment at the University of Gothenburg, Sweden. The participants were asked to make a hypothetical choice between a regular chocolate at the cost of 20 SEK (approx. €20), and a carbon-compensated “green” chocolate bar at the cost of 50 SEK (approx. €50).

### 5.2 Criterion-related validity

To test criterion-related validity, binary logistic regression was performed with purchase choice (regular vs. green chocolate) as the dependent variable and the seven dimensions of CMS as independent variables. The model correctly explained 74.2% of the choices, and Cox & Snell R2 as well as Nagelkerke R2 were satisfactory (.30 and .40 respectively), suggesting that the dimensions explain choice well. Three of the dimensions were significantly related to the choice of green chocolate. Ethics and Stimulation increases the likelihood of choosing green over regular; *B*_Ethics_=1.20, *p*<.001; *B*_Stimulation_=.61, *p*=.002; whereas Value for Money decreases the likelihood; *B*_VfM_=−1,16, *p*<.001).

For an increase of 1 unit in the Value for Money dimension, the likelihood of choosing green chocolate decreased by a factor of 3.19, while each increase of 1 in the Ethics dimension increased the likelihood by a factor of 3.32.
